# Sediment DNA Records the Critical Transition of Bacterial Communities in the Arid Lake

**DOI:** 10.1007/s00248-024-02365-4

**Published:** 2024-05-09

**Authors:** Yang Hu, Jian Cai, Yifu Song, Guoqiang Li, Yi Gong, Xingyu Jiang, Xiangming Tang, Keqiang Shao, Guang Gao

**Affiliations:** 1 Taihu Laboratory for Lake Ecosystem Research, State Key Laboratory of Lake Science and Environment, Nanjing Institute of Geography and Limnology Chinese Academy of Sciences, Nanjing, 210008 China; 2 Xiangyang Polytechnic, Xiangyang, 441000 Hubei Province China; 3 Nanjing Forestry University, Nanjing, 210008 China; 4 Lanzhou University, Lanzhou, 210008 China

**Keywords:** Alternative states, Critical transition, Sedimentary DNA, Stability, Species interactions

## Abstract

It is necessary to predict the critical transition of lake ecosystems due to their abrupt, non-linear effects on social-economic systems. Given the promising application of paleolimnological archives to tracking the historical changes of lake ecosystems, it is speculated that they can also record the lake’s critical transition. We studied Lake Dali-Nor in the arid region of Inner Mongolia because of the profound shrinking the lake experienced between the 1300 s and the 1600 s. We reconstructed the succession of bacterial communities from a 140-cm-long sediment core at 4-cm intervals and detected the critical transition. Our results showed that the historical trajectory of bacterial communities from the 1200 s to the 2010s was divided into two alternative states: state1 from 1200 to 1300 s and state2 from 1400 to 2010s. Furthermore, in the late 1300 s, the appearance of a tipping point and critical slowing down implied the existence of a critical transition. By using a multi-decadal time series from the sedimentary core, with general Lotka-Volterra model simulations, local stability analysis found that bacterial communities were the most unstable as they approached the critical transition, suggesting that the collapse of stability triggers the community shift from an equilibrium state to another state. Furthermore, the most unstable community harbored the strongest antagonistic and mutualistic interactions, which may imply the detrimental role of interaction strength on community stability. Collectively, our study showed that sediment DNA can be used to detect the critical transition of lake ecosystems.

## Introduction

Limnologists have long been fascinated by the critical transition of lake ecosystems. After the initial proposal by Holling [1], the conception of tipping points, alternative stable states, and abrupt changes have spread throughout limnological research and management. The critical transition is often defined as an ecological process in which a small disturbance suddenly triggers the ecosystem into an alternative stable state after a tipping point [2]. It is this “small changes bring big effects” pattern that makes the critical transition important for lake ecosystems. However, the detection of critical transition is particularly challenging because of the need for a long time series of high-resolution data. Yet, most studies are designed for cross-sectional observations, which do not meet this requirement [3, 4]. Thanks to advances in paleolimnology, sediment archives can extend our temporal perspective on multi-decadal timescales, tracking the historical changes of lake ecosystems [5]. In particular, a wealth of studies has suggested that the microbial communities reconstructed from sediment DNA can reveal the historical evolution of lake ecosystems [6, 7]. However, the robust proof is lacking about whether the microbial community from sediment DNA can detect the complex behavior of a critical transition.

Critical transition is theoretically announced in advance by a phenomenon known as critical slowing down (CSD), referring to the fact that the return time to the previous state increases strongly as the ecosystem moves toward the tipping point [8]. Practically, this phenomenon could be captured by several early warning signals (EWS), including amplified autocorrelation and variance in the fluctuation of the ecosystem state [9, 10]. Such measures of EWS have been applied to detect statistical signals of critical transitions from phytoplankton to zooplankton to fish communities*.* [11, 12]. Charms of the EWS-based CSD techniques are their generality and non-case-specificity; however, they only inform us about the dynamic behavior of system processes, not the diagnostic information about the underlying mechanism(s) [13]. To complement the application of CSD, recent studies perceive the critical transition as a dynamic system in which the equilibrium of biotic communities loses stability before being replaced by another equilibrium [14–16]; thus, the crux of critical transition lies in community stability [17]. However, this perspective is usually difficult to prove due to the different criteria for stability, such as resilience, resistance, persistence, and robustness [18–20].

Inspired by the pioneering work of Robert May [21], the ecological stability of microbial communities is explored by fitting mathematic models with long-time series data. The most evoked model is the generalized Lotka-Volterra (gLV) model, which describes the dynamics of a population of *N* species as a function of their intrinsic growth rates and species interactions [22]. Based on this, the stable microbial community is equivalent to the fixed point in the phase space, which is determined by the dominant eigenvalue of the Jacobian matrix: The microbial community is stable if all the eigenvalues’ real parts are negative [16, 20]. This method, known as local stability analysis, has the benefits of being both extremely general, and it is able to analyze communities with numerous species [23]. Furthermore, the gLV model can directly assess interactions between pairs of species, which influences community stability [24]. Commonly, disentangling species interactions is built on correlation networks – identifying species that occur together more often than expected by chance alone [25, 26]. However, this method may introduce biases, particularly in highly diverse communities [27], producing confusion about the effects of species interactions on stability [23, 28–30]. For instance, if two species frequently co-occur, it is hard to conclude whether they facilitate one another by cooperation or occupy a similar niche by competition. Therefore, much current research infers species interactions through the gLV model, which investigate beyond the co-occurrence to scrutinize how species change in response to one another. In this way, Mounier and colleagues (2008) [31] modeled cheese fermentation community interactions, which predicted negative interactions that were afterward confirmed by co-culture experiments. Similarly, Stein et al. (2013) also quantified species interactions of the human gut microbiome after antibiotic perturbations [32].

Collectively, characterizing the dynamics of microbial communities through the gLV model offers a window into their critical transition from the perspective of stability and species interactions. Empirically, we reconstructed the historical dynamics of bacterial communities in a sediment archive through a gLV model. By this approach, we addressed three major questions: (1) whether the critical transition of microbial communities could be detected by sedimentary DNA; (2) if so, how does the community stability change during the critical transition; and (3) what is, or are, the underlying mechanism(s) by which species interaction affects community stability. Together, our results provide an unprecedented picture of the critical transition in paleolimnological records and also provide evidence to support that studying sediment DNA is an essential tool for understanding community dynamics in lake ecosystems.

## Materials and Methods

### Study Site, Sampling, and Core Chronology

**Fig. 1:**
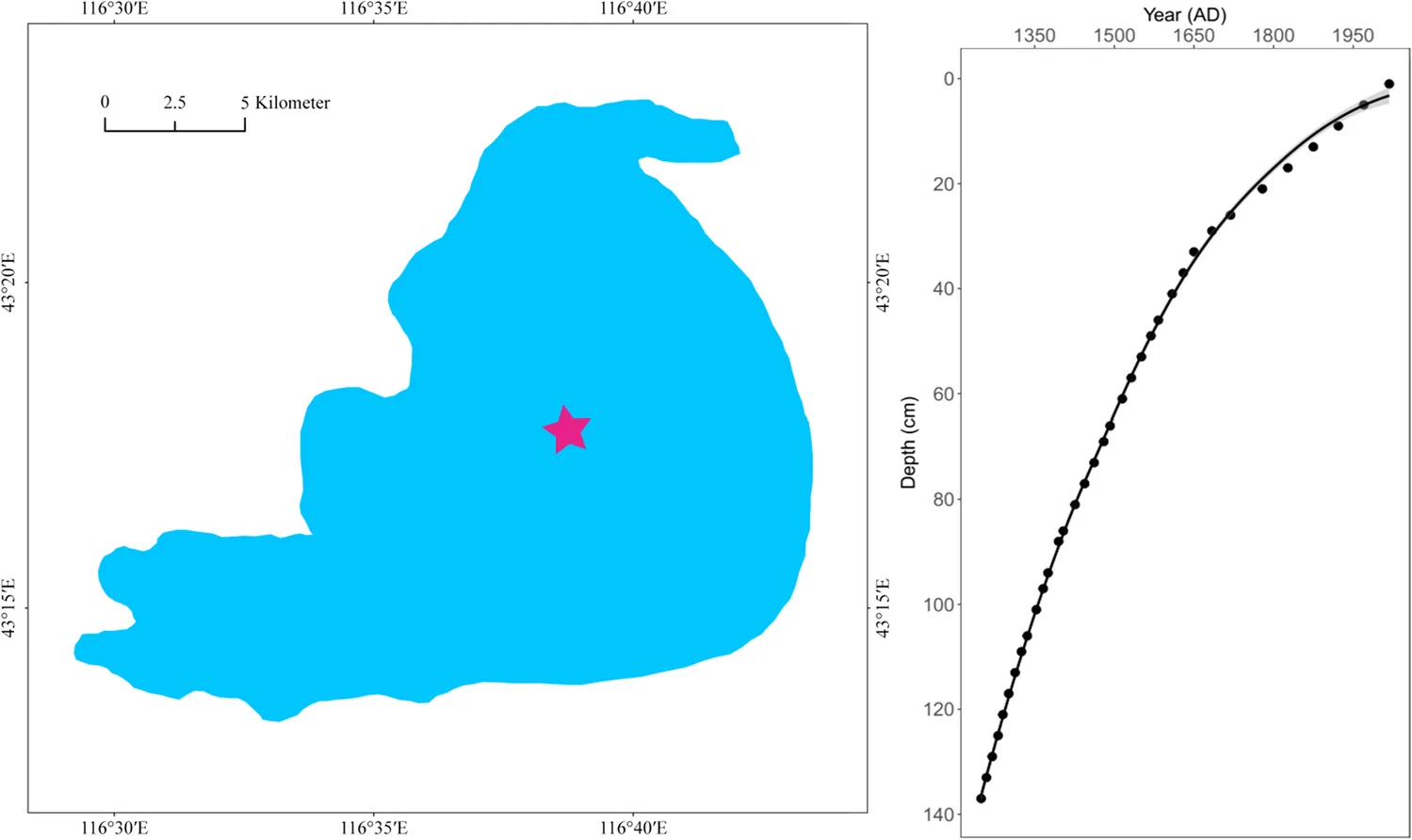
Sampling site of Lake Dali-Nor (left) and the age of sediment core with depth (right)

Lake Dali-Nor (43°13′–23′ N, 116°29′–45′ E, ~ 1230 m above sea level) is an inland closed-basin lake in central Inner Mongolia, which has an area of 238 km^2^ and an average depth of 10 m. Given its location at the modern limit of the East Asian summer monsoon, the lake sedimentation process is highly sensitive to climate change, suggesting that the sediments of Lake Dali-Nor provide valuable paleo-climate archives [33]. We choose our exact study site (43°16.68′ N, 116°37.26′, Fig. 1) because its sedimentation rates and visible lamination have been well characterized by Xiao et al. (2008) [34]. A 137-cm uncontaminated sediment core was collected in the center of the lake in October 2020 using a gravity multi-corer. The core was split and cut at 4-cm intervals on average, which produced 35 sub-samples. They were then bagged in labeled Whirlpak bags on the day of field coring and refrigerated until laboratory analyses. Half of each sub-sample was sieved (100-μm mesh) after drying. The total sediment samples were disposed for accelerator mass spectrometry (AMS) radiocarbon dating. We compounded the graphite in the ^14^C dating laboratory in Lanzhou University by the normative approach and dated by accelerator mass spectrometry (AMS) in Peking University. All ^14^C ages were recalibrated to calendar years using the IntCal20 calibration curve [35].

### Molecular Analyses

The total microbial genomic DNA was extracted from sediment samples by the PowerSoil DNA Isolation Kit (Mo Bio Laboratories, Inc., Carlsbad, CA, USA). To determine the 260/280 nm and 260/230 nm absorbance ratios, we used a NanoDrop ND-2000 spectrophotometer (NanoDrop, ND-2000, Thermo Scientific, 111 Wilmington, DE, USA). The V4 hypervariable region of the 16S rRNA gene was amplified to assess the bacterial community using the primers 515F (5’-GTGYCAGCMGCCGCGGTAA-3’) and 806R (3’-GGACTACNVGGGTWTCTAAT-5’). As prior study suggested, older sediment layers favor the shorter fragments, we chosen 2 × 250 bp paired-end sequencing to extract the bacterial communities in sediment core [36].

The raw data for pools of samples were separately trimmed and de novo assembled in a unique file using CLC Genomics Workbench (Version 6.0.2, CLC Bio, Denmark) alignment and annotation tools. We removed sequences if they (1) contained more than one ambiguous nucleotide, (2) lacked a complete barcode and primer at one end, or (3) were shorter than 200 bp after removal of the barcode and primer. The overlap settings for this assembly were a mismatch cost of 2, an insert cost of 3, a minimum contig length of 200 bp, a similarity of 0.8, and a trimming quality score of 0.05. The sequences were clustered at 97% similarity cutoff into operational taxonomic units (OTUs). The representative sequence of each phylotype was aligned against the SILVA database (release 132) with a confidence threshold of 80%. All archaeal and chloroplast sequences were removed before downstream analysis.

### Model Analyses

To simulate the dynamics of bacterial communities, we adopted the well-known gLV model:

1 $$\frac{d{N}_{i}}{dt}= {N}_{i}\left({r}_{i}- \sum_{j=1}^{S}{a}_{ij}{N}_{j}\right)$$

where *N*_*i*_ is the relative abundance of class-level group *i, i* = 1, …,* S*, *r*_*i*_ is its intrinsic growth rate, and *a*_*ij*_ is the interaction strength that captures how strongly class *j* inhibits class *i*. One of the key features of this model is the ability to consider three types of biotic interactions: mutualism (both *a*_*ij*_ and *a*_*ji*_ are positive, + / +), antagonism (both *a*_*ij*_ and *a*_*ji*_ are negative, − / −), and exploitation (*a*_*ij*_ is positive and *a*_*ji*_ is negative, and vice versa, + / −).

By reconstructing Eq. (1) and applying forward difference to the time-continuous dynamical system, the following discrete system of equations was obtained.

2 $$\frac{\Delta {\mathrm{ln}}{N}_{i}\left({t}_{k}\right)}{\Delta {t}_{k}}={r}_{i}-\sum_{j=1}^{S}{a}_{ij}{N}_{j}\left({t}_{k}\right)$$

Based on this linear system, the bacterial composition data we extracted from the sediment core can be used to solve the parameters ${r}_{i}$ and ${a}_{ij}$. To ensure the stability of the solution, we implemented Tikhonov regularization in this work, coupled with the application of *k*-fold cross-validation, in an effort to achieve the best predictive performance. Taking into account both the complexity of the model and the predictability of the unknown parameters, this kind of regularization approach can effectively reduce the risk of overfitting.

Moreover, we analyzed the steady states of the dynamic system (1) which refers to the states where ${N}_{i}$ no longer change over time, i.e.,

$${f}_{i}\left({\boldsymbol{N}}\right)={N}_{i}\left({r}_{i}-\sum_{j=1}^{S}{a}_{ij}{N}_{j}\right)=0, i=1,\dots ,S.$$

The spectrum distribution of the Jacobian matrix ${\left(\partial {f}_{i}/\partial {N}_{k}\right)}_{i,k}$ in the steady states can provide an approach for qualitative analysis of stability. According to the principle of linearized stability, if the real part of the eigenvalues of the Jacobian matrix are all negative, the states are asymptotically stable. If they are not, the states are unstable.

### Statistical Analyses

All analyses and visualizations were performed by R environment (Version 3.2.2, http://www.r-project.org) and Matlab (version 2021b).

For the 16S rRNA gene amplicon sequencing data, rarefied OTU tables were created according to the minimum number of reads among all samples. The relative abundance of specific phylogenetic groups was assessed as the number of sequences affiliated with that group divided by the total number of sequences per sample. The alpha diversity was represented by Richness, Shannon, and Simpson indices. The statistical differences of each index were tested by non-parametric Kruskal–Wallis test. To determine the beta diversity of the bacterial community, we performed Bray–Curtis dissimilarity-based non-metric multidimensional scaling (NMDS) analysis. To statistically test the difference in bacterial community composition, we performed an analysis of similarity (ANOSIM) and PERMANOVA with 999 permutations. The ANOSIM then generated a test statistic *r*, with a score of 1 indicating complete separation and 0 indicating no separation. By assuming the future will resemble the past [13, 37], an ARIMA-based model based on the community state (score of NMDS1) was identified by autoregressive integrated moving average, with the R function arima (*p*, *d*, *q*), in which *p* is the autoregressive order, *d* is the degree of differencing, and *q* is the moving-average order. To identify the major change moment of alpha-diversity in the time series, we applied structural-change analysis the R package *strucchange* through minimizing the residual sum of squares in multiple regression segments.

### Data Deposition

The raw sequence data reported in this paper have been deposited in the Genome Sequence Archive (Genomics, Proteomics, and Bioinformatics 2017) of the BIG Data Center, Beijing Institute of Genomics (BIG), Chinese Academy of Sciences, under the accession number CRA011047. The data are publicly accessible at https://bigd.big.ac.cn/gsa.

## Results

### Historical Succession of Microbial Communities through the Sedimental Core

**Fig. 2:**
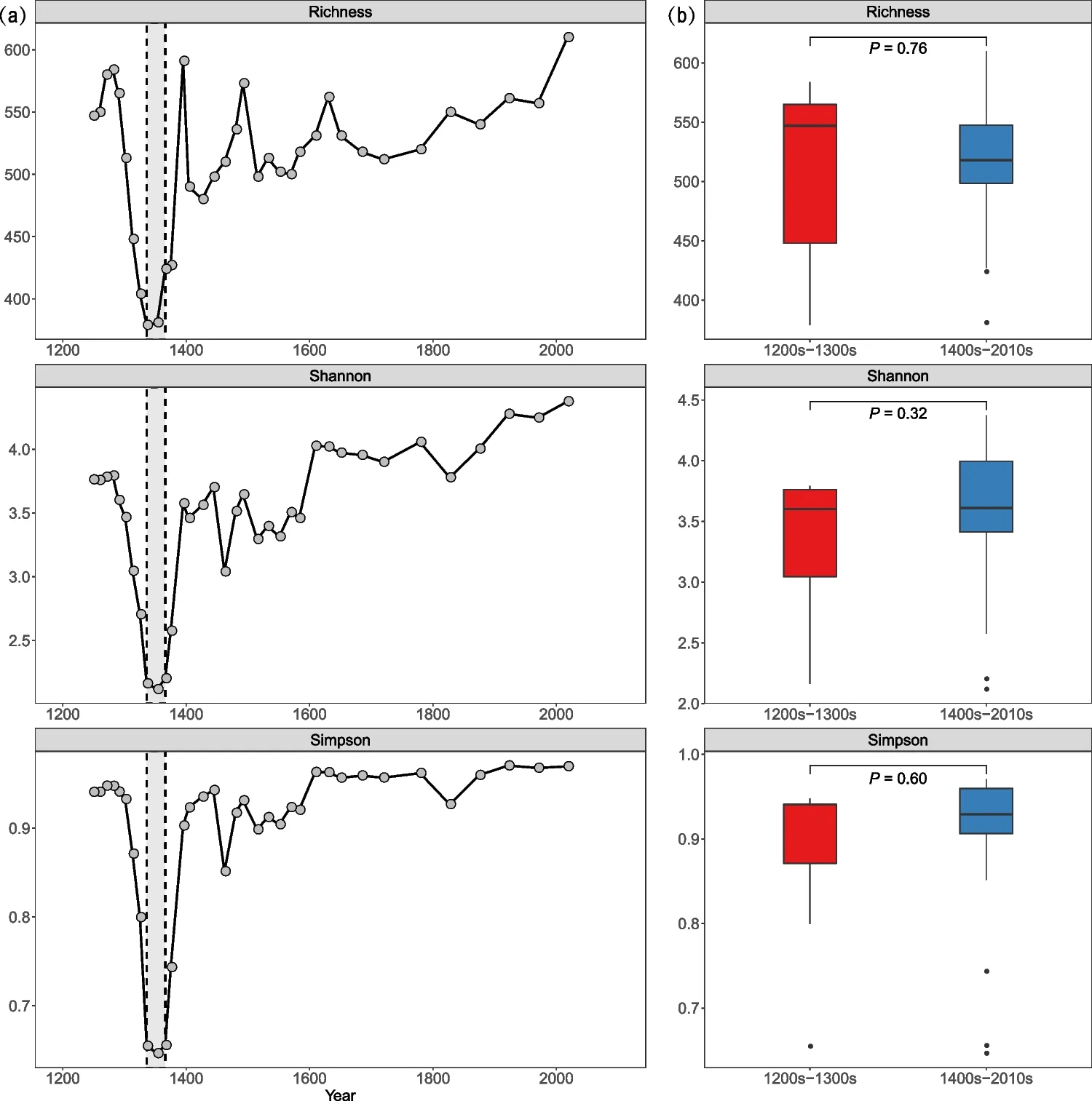
Alpha diversity of bacterial communities over the sedimental core. (**a**) The temporal change of Richness, Shannon, and Simpson from 1200 to 2010s. (**b**) The comparison of Richness, Shannon, and Simpson between 1200 s–1300 s and 1400 s–2010s

After all the filtering and processing steps applied for sequencing raw data, 77,885 high reads were produced and were clustered into 1828 OTUs, with 814 ± 58 OTUs per site. Alpha diversity, as expressed by Richness, Shannon, and Simpson indices, temporally experienced a major breakpoint in years between 1336 and 1366: Each index sharply decreased and then immediately increased in the vicinity of the breakpoint (Fig. 2a). After the late 1300 s, they oscillated and eventually reverted to their original levels (Fig. 2b, all *P* > 0.05).

**Fig. 3:**
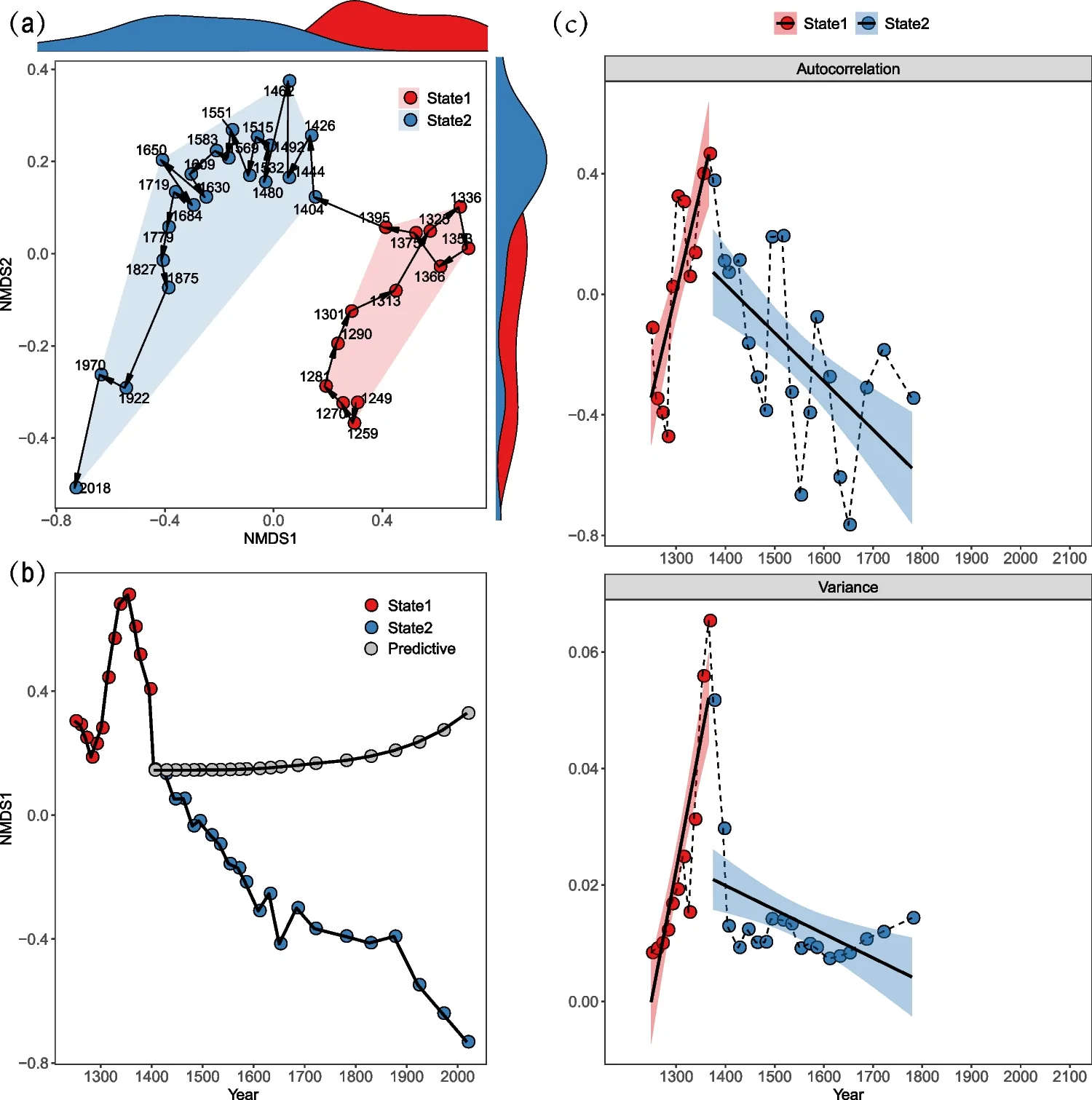
Beta diversity of bacterial communities through the sedimental core. (**a**) Non-metric multidimensional scaling (NMDS) analysis of bacterial communities; (**b**) the ARIMA (1, 1, 0) forecast derived from state1; solid lines show the observed values, and dashed lines show the predictive values. (**c**) Lag-1 autocorrelation and variance of NMDS1 scores of bacterial communities with a sliding window of 5 core samples

The beta diversity of the bacterial communities also indicated an abrupt change in the late 1300 s during their historical succession. The stratigraphically constrained cluster analysis based on the Bray–Curtis distance clearly divided the succession into two distinct states (ANOSIM test, *P* < 0.001): the years from 1200 to 1300 s and years from 1400 to 2010s. By interpreting the ordination plot as a representation of the phase space, the historical trajectory of bacterial communities showed a unidirectional, non-replacement succession, with little overlap between these two states (Fig. 3a). Furthermore, the frequency distribution of states (the Gaussian kernel density estimation) for the periods 1200 s–1300 s (red) and 1400 s–2010s (blue) showed a bimodality of bacterial communities (Fig. 3a), suggesting the classic bistable system. Together, the significant differences in the bacterial communities between the two states implied that the critical transition occurred in the late 1300 s. To verify this implication, we forecast the status response of bacterial communities in the state2 by the ARIMA model derived from the state1 and showed that the predicted values significantly deviated from observed ones (Fig. 3b). Furthermore, we also scrutinized two EWSs to detect the CSD phenomenon: Lag-1 autocorrelation and variance of NMDS1 scores, which significantly increased from the 1200 s to the 1300 s and subsequently declined until the 2010s (Fig. 3c).

**Fig. 4:**
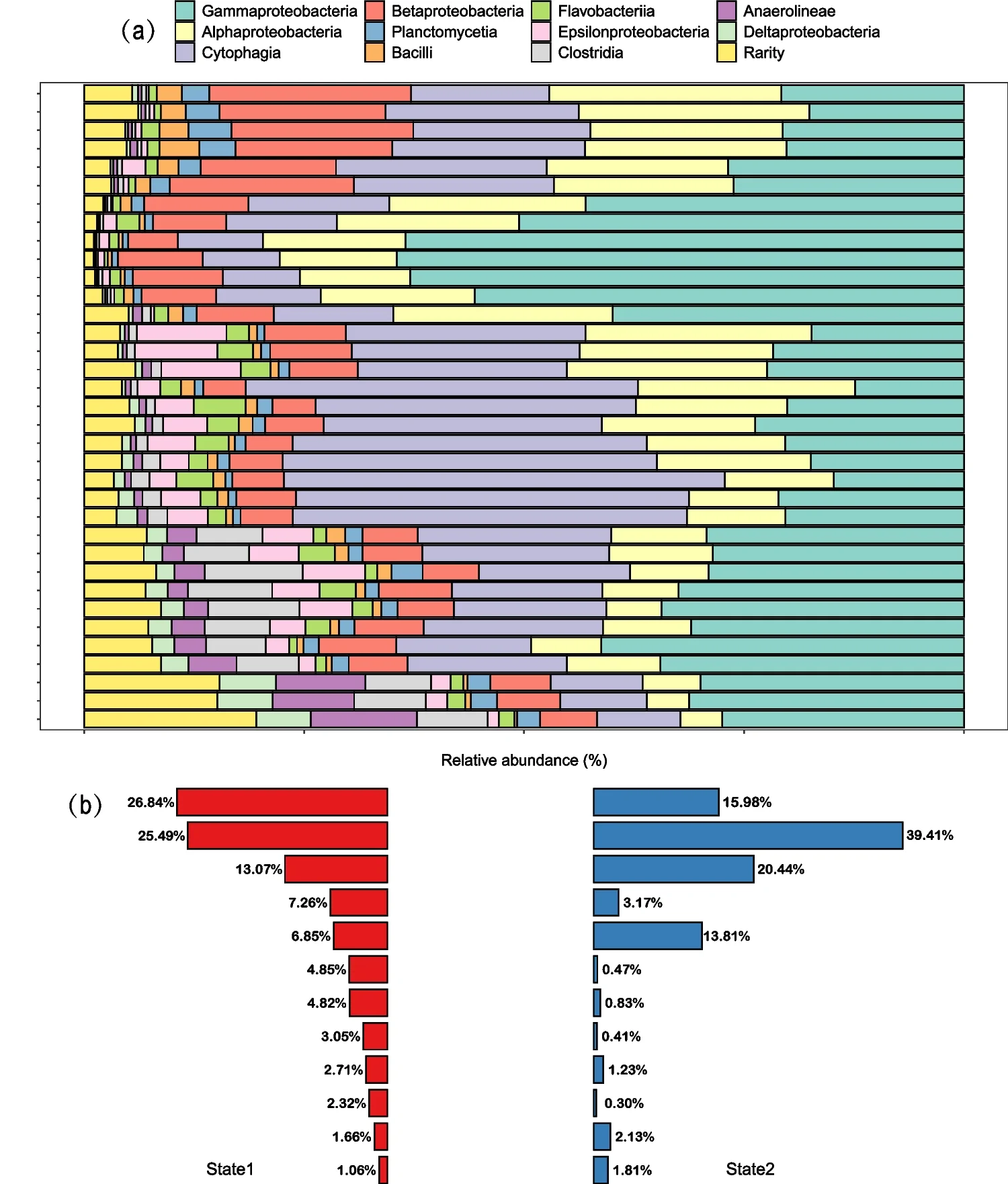
The composition of bacterial communities through the sedimental core, showing 12 classes. (**a**) Profile of bacterial communities from the 1200 s to the 2010s; (**b**) comparison of relative abundance of classes between state1 and state2

Most of the OTUs could be classified into a defined taxonomic group: 97.92% were classified into 92 bacterial class-level taxonomic groups. During the historical succession, the four dominant bacterial classes (with relative abundance > 1% in each sample) were Gammaproteobacteria, Cytophagia, Alphaproteobacteria, and Betaproteobacteria, which accounted for approximately 80% of the whole community, respectively (Fig. 4a). Although these four classes numerically prevailed in bacterial communities in both states, their dominance ranks were different between two states (Fig. 4b). Specifically, in state1, the dominant classes (in descending order) were Cytophagia, Gammaproteobacteria, Alphaproteobacteria, and Betaproteobacteria, whereas in state2, the dominant classes were Gammaproteobacteria, Alphaproteobacteria, Cytophagia, and Betaproteobacteria (Fig. 4b).

### Species Interactions and Community Stability during the Historical Succession

**Fig. 5:**
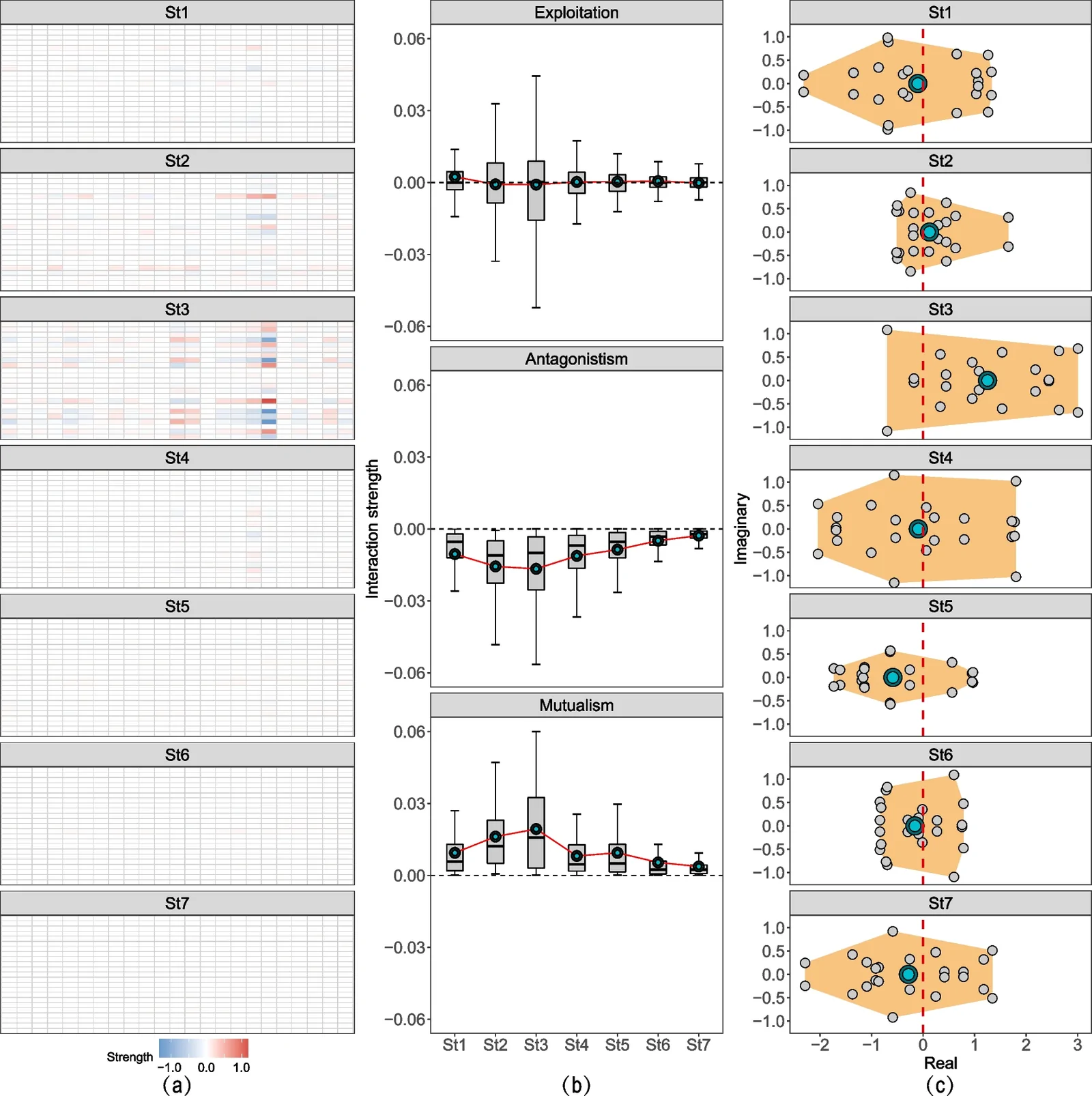
Species interaction and community stability from stage1 to stage7 (St1–St7). (**a**) Interaction strength in row i and column j represent the effect of class j on i, where red stands for activation and blue for repression. (**b**) Strength of three interaction types: exploitation, antagonistism, and mutualism, respectively. (**c**) Eigenvalue distribution of Jacobian matrix for the equilibrium point. In (b) and (c), the blue circles are the average value

By choosing a window length of 10 cores and a step size of 5 cores (half the window length), bacterial communities through 35 sediment cores were discretely split into 7 groups, which are representative of the 7 stages (stage1–stage7) during the succession from stage1 to state2. The gLV model inferred the change pattern of species interactions from stage1 to stage7 (Fig. 5a). Firstly, the results showed that the proportion of exploitative interactions (43.82%) significantly overwhelmed that of antagonism (29.93%) and mutualism (26.25%) (*P* < 0.01). Despite the dominant role of exploitation in the species interactions, their interaction strength was generally around zero during the whole succession, whereas the strength of both antagonistic and mutualistic interactions increased from stage1 to stage3 and decreased from stage3 to stage7 (Fig. 5b). Secondly, the gLV model also inferred the community stability by assessing the real part of eigenvalue of the Jacobian matrix (Fig. 5c). The results demonstrated that the bulk eigenvalues contained a mixture of negative and positive values. By regarding the centroid of bulk eigenvalues as the mean stability level, the real parts of eigenvalue were negative in stage1 and passed to positive values in stage2–stage3 and then rebounded to negative values in stage4–stage7.

## Discussion

### Bacterial Communities Show the Critical Transition during the Historical Succession

Due to regional drying and decreased precipitation, Lake Dali-Nor has experienced profound shrinking between the 1300 s and 1600 s [34, 38]. Such water imbalance is expected to dramatically alter the ecosystem structure and function. However, it has been unclear whether there was a critical transition of Lake Dali-Nor during this period. By our reconstruction of the historical succession of bacterial communities derived from the sediment-core DNA in Lake Dali-Nor from 1200 to 2010s, we show that these communities did indeed exhibit a critical transition in the late 1300 s.

Recently, the successful application of paleolimnological records in tracking the temporal changes of the lake state suggested that the biotic imprints are able to detect the critical transition [39]. For instance, Ibrahim et al. (2020) revealed two abrupt transitions of microbial eukaryotes, diatoms and cyanobacteria during the eutrophication of Lake Constance in Germany [6]. Similarly, Sagrario et al. (2020) determined the critical transitions of multiple-trophic proxies in Lake Blanca Chica (Argentina) [40]. Together, these results highlight the paleolimnological evidence for a critical transition in lake ecosystems [7, 41]. However, most relevant studies identify the critical transition only by significant distinctness between communities according to sequential algorithms and statistical tests, such as the Sequential T-test Algorithm for Analyzing and the F-statistic [42, 43]. Apparently, these methodologies largely neglect the non-linear response of biotic communities to external disturbances. In addition, sediment-core records also have their own biases and shortcomings, such as sedimental mixing, irregular temporal integration, and DNA degradation, resulting in that paleolimnological data series are not strictly analogous to experimental monitoring data sets [44]. To address these limitations, we provide a series of judgments to promote the robustness and significance of the critical transition detected in Lake Dali-Nor.

Theoretically, there is a recognition that the critical transition is a re-organization of biotic communities by producing a new state [45]. Our results found that the temporal succession of bacterial communities in Lake Dali-Nor was divided into two significantly distinct states: state1 from the 1200 s to the 1300 s and state2 from the 1400 s to the 2010s. By interpreting the ordination plot as the phase space [18], the bimodality along NMDS1 and the complete separation between the dispersion of two community groups imply two alternative states of bacterial communities. Although this ordination plot-based method seems intuitive, it can, to some extent, serve to depict the behavior of dynamic communities. For instance, the ordination plot of the human gut microbiome from four countries visualizes three attractors, suggesting that there are three states of the human gut microbiome [46]. In addition, the critical transition of bacterial communities also undergoes dramatic shifts in dominated groups between alternative states. Primarily, the dominance rank of bacterial communities was re-structured between state1 and state2: *Cytophagia* was the dominant group before the transition, yet dropped to being only the third most dominant group after the transition; in contrast, *Gammaproteobacteria* and *Alphaproteobacteria* were ranked second and third before the transition and rose to be ranked first and second after the transition. Moreover, the increase in increasing *Gammaproteobacteria* and *Alphaproteobacteria*, as typical halophilic bacteria, corresponds with the shrinking of Lake Dali-Nor after the 1300 s, when lake shrinkage commonly leads to higher salinity [47]. Collectively, the significantly distinct traits at both the community level and the class level after the late 1300 s indicate two alternative states of bacterial communities during the historical succession.

However, the appearance of alternative states alone is not sufficient to identify the critical transition of bacterial communities. Another hallmark of critical transition is the non-linear shift between alternative states [2, 48]. To verify the detected critical transition of bacterial communities during their historical succession, we therefore provided supportive pieces of evidence of the tipping point and the CSD phenomenon [49, 50]. First, we found a major breakpoint between 1336 and 1366 in the change of alpha diversity, which coincides with the period of the late 1300 s when the critical transition was detected. The dramatical decrease in alpha-diversity may stem from that the bacterial species adapted to the original environmental set of the previous alternative state would suffer intensive stresses as they settled into a new environment of the later alternative state. By assuming this breakpoint as the tipping point, it is expected that the bacterial communities would jump abruptly to another succession trajectory [51]. Consistent with this assumption, the ARIMA model showed that the predicted community of state2 (NMDS1) deviated from the observed values, implying that there was a bifurcation of the bacterial communities at this breakpoint. Furthermore, a wealth of theoretical and empirical studies shows a CSD phenomenon in biotic communities as they approach the critical transition, which is primarily marked by variance and autocorrelation of community status [8, 10]. In our study, we also found that both variance and autocorrelation significantly increased until the late 1300 s, which reflects the bacterial communities become more correlated to their past and are pushed stochastically in the vicinity of bifurcation. Note, although the rising variance and autocorrelation have been previously reported during the paleo-climate transition and lake eutrophication [6, 52], it is not to say that it is flawless in their practice. One of the major shortcomings is the difficulty in determining the casual mechanisms: whether the environmental factors fluctuate stochastically, or whether the communities have reduced capacity to follow high-frequency environmental fluctuations [9, 53]. Nevertheless, our findings that two alternative states were bridged by the tipping point and the CSD phenomenon do infer the critical transitions of bacterial communities during their historical succession.

### Strong Interaction Strength Disrupts the Stability of Bacterial Communities

It is predicted that the biotic community is progressively unstable prior to the tipping point, suggesting that the collapse of community stability triggers the critical transition [54]. Although this hypothesis is esthetically appealing, one major obstacle in confirming this is the multi-faceted definition of community stability, ranging from the persistence of whether a community maintains the species with which it started [55] to feasibility which focuses on the coexistence of all species [56], and to the level of variability displayed by the community over time [57]. Conspicuously, these definitions largely limit community stability to the community composition. In our present study, we determined the community stability based on the equilibrium population dynamics by fitting the sequencing data into the gLV model. The results showed that the real parts of the eigenvalue of the Jacobian matrix were a mixture of negative and positive values, suggesting that the equilibrium states of bacterial communities are not equivalent to the fixed point in phase space. Indeed, the equilibrium state of biotic communities in nature actually performed as chaos, periodicity, and a limit cycle [58]. Thus, we have here defined stability as the likelihood that the community converges to a feasible equilibrium state, which is quantitatively evaluated by the average value of real parts for all eigenvalues [16, 23]. By this criterion, we found that the earlier and later succession stages (stage1 and stage4–stage7) were stable, whereas the intermediate regime (stage2–stage3) was unstable, which signifies that the critical transition consists of two alternative stable states separated by an intermediate unstable state. Consistently, this implication also fits the prediction of dynamical system theory, in which the dynamic shifts of microbial communities are a continuum of shifting between stability and instability [17]. Furthermore, the largest positive eigenvalue is in stage3, which contains the detected tipping point, suggesting that bacterial communities in the tipping point are the most unstable, echoing that the collapse of stability causes a critical transition [54, 59, 60].

Microbes do not exist in isolation, but reside with myriad individuals through complex interactions [61]. Recently, a large body of studies has found that interspecific interactions strongly act on community stability [62]; however, the heterogeneity of results led to multiple different understandings about the roles of interspecific interactions. For instance, some researchers suggest that competition destabilizes microbial communities [28], yet some others argue that competition benefits the stability of microbial communities [23, 63]. Similarly, contrasting roles of cooperation in community stability have also been documented [16, 20]. These mixed conclusions about the effect of species interactions on stability largely stem from two limitations: (1) Detecting causal interspecific interactions is not straightforward because manipulative experiments are not feasible when numerous species and interactions are targeted under field conditions [27]; and (2) most previous studies assumed the pairs of species symmetrically interact by antagonistic (− / −) and mutualistic interactions (+ / +), but neglect the asymmetrical exploitations (+ / −) in which one species tends to gain a fitness benefit at the expense of another [64, 65]. To address these questions, our study therefore accessed how one species interact with another by applying the gLV model. Strikingly, results showed that the proportion of asymmetrical interactions significantly overweighed symmetrical interactions during the historical succession of bacterial communities. After a careful literature review, however, we found few relevant studies with which we could compare our work, which also indicates this method is still in its infancy and more rigorous exploration is needed.

Nevertheless, this is a new approach to the study of species interactions and the stability of bacterial communities. By incorporating the key biologically realistic assumption of asymmetrical exploitations, we found that antagonistic and mutualistic interactions had the strongest strength in the period of stage3 when bacterial communities were the most unstable. Consistent with this, previous evidence has also found that the intensifying competitive interaction of bacterial communities gradually decreased community stability [28]. Furthermore, a computational framework also predicts that increasing interaction strength transitions the microbial ecosystem from a stable equilibrium to a persistent fluctuating state by expanding the competitive interaction to cooperative interaction [22]. The detrimental role of strong interaction strength on bacterial communities could be explained by two reasons. First, increasing the strength of mutualistic interactions can increase the dependency among species so that if one species fluctuates, the strong positive feedback will amplify the causal responses of another species [23]. Second, the higher antagonistic interactions are expected to exclude species from the community, leading to partial extinction and a less stable state [66, 67]. In our current study, we speculate that the bacteria of Lake Dali-Nor displayed a trade-off between interaction strength and community stability to promote their survival in the higher salinity habitat after the 1300 s.

## Conclusion

Since the Anthropocene, lake ecosystems have experienced an abrupt, non-linear critical transition; however, it has been unclear whether sediment DNA could record this critical transition. By using a multi-decadal time series from the sediment core with the gLV model simulations, we detect a critical transition of bacterial communities in Lake Dali-Nor that occurred in the late 1300 s. We showed that the historical succession was divided into two distinct alternative stable states, which were punctuated by an intermediate unstable state. Furthermore, the most unstable community harbored the strongest antagonistic and mutualistic interactions, which may imply the detrimental role of interaction strength on community stability. Collectively, our results show that paleolimnological evidence holds considerable promise for studying the critical transition of lake ecosystems.

## Data Availability

The raw sequence data reported in this paper have been deposited in the Genome Sequence Archive (Genomics, Proteomics, and Bioinformatics 2017) of the BIG Data Center, Beijing Institute of Genomics (BIG), Chinese Academy of Sciences, under the accession number CRA011047.
